# Classifying Foot Arch Morphologies Using High-Sensitivity Pressure Mat Technology: Prevalence Estimates and Associations With Pain, Activity Limitations, and Patient Characteristics in a Community-Based Adult Sample

**DOI:** 10.7759/cureus.114287

**Published:** 2026-08-10

**Authors:** J. Benjamin Jackson, Harley T Davis, Rajat Das Gupta, Nathan Delahunty, Travis Dias, Heidi C Ventresca, Tyler A Gonzalez, Anthony J Alberg

**Affiliations:** 1 Orthopedic Surgery, University of South Carolina, Columbia, USA; 2 Orthopedic Surgery, Prisma Health Midlands, Columbia, USA; 3 Epidemiology and Biostatistics, University of South Carolina, Columbia, USA

**Keywords:** analysis of foot position, arch contact, flatfoot, foot pain, high arch foot

## Abstract

Introduction

There is a lack of consensus in the literature regarding the clinical understanding of foot arch contact variations in the general population, as most studies have focused on specific demographic groups or risk factors. This limits the ability of clinicians to communicate risk and interpret arch-related findings in diverse patient populations. This cross-sectional study evaluated the use of a novel pressure mat method to categorize adults as having flatfoot, high arch, or normal feet in a large convenience sample and examined demographic and medical characteristics associated with these arch variations.

Methods

A high-sensitivity pressure mat measured midfoot contact areas during static bipedal stance in 1,015 adults (2,030 feet). Feet were categorized as flat (midfoot area > mean + 1 SD), high arch (midfoot area < mean - 1 SD), or normal (± 1 SD). Demographics, comorbidities, activity limitation, and foot pain were collected via medical records and surveys. Associations were analyzed using chi-square tests and multivariable logistic regression, adjusting for age, gender, race/ethnicity, education, income, activity level, BMI, hypertension, diabetes, and arthritis.

Results

Prevalence was 11.1% (113 participants) for at least one flatfoot (36, or 3.5%, bilateral) and 18.3% (186 participants) for at least one high arch (88, or 8.7%, bilateral). Flatfoot was significantly associated with male sex (AOR = 5.0, 95% CI: 2.8-8.9), African American race (AOR = 5.7, 95% CI: 3.0-10.8), other race (AOR = 3.8, 95% CI: 1.2-12.3), obesity (AOR = 5.1, 95% CI: 1.7-15.6), and arthritis (AOR = 2.0, 95% CI: 1.2-3.6); high arch showed inverse associations. Participants with flatfoot reported significantly higher rates of activity limitation (27, or 24.3%) and foot pain compared to normal-arch participants, while high-arch participants reported less pain and more activity than those categorized as having normal arches.

Conclusion

Using a high-sensitivity pressure mat, we estimated flatfoot prevalence at 11.1% and high-arch prevalence at 18.3% in a community-based adult sample. Foot morphology categories demonstrated significant associations with demographics, comorbidities, and, for flatfoot, activity limitations and foot pain, both aligning with and extending prior literature. To our knowledge, this study is among the first to apply pressure mat technology for arch classification in a large, diverse sample, suggesting potential utility for clinical screening and risk communication using these estimated baseline measures. Pressure mats may offer a rapid, non-invasive screening tool for foot arch assessment, though validation against clinical examination is needed before clinical implementation.

## Introduction

A consensus on a scientific definition for 'normal' arch contact does not exist. While clinical patterns such as cavovarus or planovalgus are recognized, the frequency, distribution, and clinical significance of arch contact variations in the general population remain incompletely characterized. This gap reflects multiple factors, including the lack of standardized measurement techniques [1-6], the use of different classification thresholds across studies [7-10], and the clinical reality that arch variations often only come to attention when they cause pain or functional limitations [11-13].

Many of these factors can be partially addressed with the use of more modern technologies, which provide extremely accurate and sensitive foot pressure and area measurements [14-18]. Newer technologies, including pressure mats, can rapidly produce arch contact determinations. While prior studies have sought to characterize normal arch contact using radiographically derived angular measurements [6,8-10,12,13], inked footprint characterizations [3,7,19,20], navicular and midfoot height measurements [1,21], and self-report [22], new technologies can provide a broader understanding of foot characteristics and pressure distributions during normal bipedal stance [15-18]. These modalities additionally permit efficient examination of arch contact in large populations, potentially leading to more accurate arch contact prevalence estimates.

The current literature on prevalence estimates for flat and high arch feet is variable. Beyond a focus on patients with clinical symptoms related to arch height [3,11,13,23], many recent studies have examined arch contact distributions in children [4,24,25] or more homogeneous populations [3,6,12,13,23]. In studies focused on adults, prevalence estimates have been inconsistent, ranging from 2.6% to 47.8% for flat feet and 5.2% to 49.6% for high arch feet [1-5,11,20,23,26-28]. This variability is likely due to small sample sizes, a focus on patients with similar characteristics, recruitment in clinics serving symptomatic patients, and different methods used to determine arch height.

Given the inconsistencies between arch height measurement methods and the lack of consensus in the literature regarding the clinical understanding of foot arch contact variations in the general population, the current study evaluated the distribution of midfoot foot area collected with a pressure mat, estimated both flatfoot and high arch prevalence in a large convenience sample of adults, and examined study participant characteristics associated with the foot arch categories. Additionally, we examined associations of participant self-reported pain and functional limitations with their arch height to help support the methodology used to categorize participant feet. To the authors’ knowledge, this is one of the first studies to utilize pressure mat readings only for the purposes of categorizing foot arch. Besides describing the distributions of flatfoot and high arch in participants, it was hypothesized that participants categorized as having flat or high arch feet would report more pain and/or limitations than those categorized as having normal feet, as has been previously described in the literature. Additionally, it was hypothesized that participant demographics and comorbidities would be associated with having flat or high arch feet. While the purpose of this study was not to validate this categorization methodology through clinical diagnosis of flat (or high arch) feet, it was instead to describe the prevalence of these conditions based on a novel measurement technique in a large convenience sample.

## Materials and methods

Study sample

Participant recruitment, enrollment, and data collection for this cross-sectional study were conducted from May to July 2021 at four community clinic/hospital locations within a single academic center. Clinics were visited on different days and at different times during the day to improve the representativeness of the convenience sample. In addition, eligible participants included patients attending the clinics, their family and friends, clinic/hospital staff, and other present and willing individuals. Individuals were eligible if they were ≥18 years of age, had both of their lower extremities, and could stand unassisted. Exclusion criteria included a history of stroke or foot drop (to exclude individuals with foot arch variations related to neurological conditions), prior surgery below the knee, or the inability to provide written consent in English. Study staff approached potential participants, screened them for eligibility, and enrolled those who consented. The academic center's Institutional Review Board (IRB) approved the study (approval no. 1852630-4), which was conducted in accordance with the STROBE guidelines.

Data collection and measurement

For each participant, a plantar pressure profile of both feet was obtained using a mobile pressure mat (Tekscan MobileMat™ model MMat-RES, sensor resolution 1.0 sensels/cm²) during static bipedal stance (Tekscan, Inc., Norwood, MA, USA). Participants stood barefoot on the mat with feet positioned approximately shoulder-width apart and were instructed to distribute weight evenly and look straight ahead. The mat was calibrated according to the participant's weight (in lbs) prior to each participant's measurement per the manufacturer's protocol. Each participant stood on the mat for 10 seconds to calibrate the mat, followed by an additional 10 seconds to collect one image of both feet. The FootMat Research 7.10 software (Tekscan, Inc., Norwood, MA, USA) was used to generate foot length (cm), peak pressure (kPa), peak contact pressure (kPa), and contact area (cm²) measurements. Contact area, peak pressure, and peak contact pressure were obtained for four (toe, forefoot, midfoot, and heel) regions on both feet, while total foot length was obtained once for each foot.

Participants consented to a review of their medical records or self-reported patient characteristics, including age, sex, race, ethnicity, body mass index (BMI; categorized as underweight/normal, overweight, or obese), and presence/absence of hypertension, diabetes, and any type of arthritis. In addition, study participants filled out a brief questionnaire at the time of enrollment that collected household income, highest education level completed, employment status, current physical activity level (inactive, slightly active, moderately active, or very active), and overall current foot pain (none, both feet, or one foot), as well as pain in the arch of the foot (none, both feet, or one foot). As an incentive for participation, participants were entered into a drawing to win a $100 gift card. During the three-month timeframe, 1,025 individuals were enrolled. Nine participants later withdrew consent, and inaccurate pressure data were noted for one participant; therefore, the final sample size was 1,015 participants or 2,030 feet.

The calculated midfoot total area for each foot was used to establish cut points and categorize feet as flat, normal, or high arch. Due to skewness in the distribution of midfoot areas in the sample, values were log-transformed prior to categorization. Mean and standard deviation (SD) of the log-transformed midfoot total area values were calculated independently for right and left feet (right: mean 3.02 ± 0.718; left: mean 2.81 ± 0.909). Cut points were then established based on the sample distribution: flatfoot was defined as midfoot total area > (mean + 1 SD), high arch as midfoot area < (mean - 1 SD), and normal as midfoot area within ±1 SD of the mean. This ±1 SD threshold was chosen based on the assumption that the normal category represented the baseline, with more extreme values above and below the mean representing abnormal (i.e., flatfoot or high-arch) foot classifications. 

Statistical analysis

For all analyses, individuals were classified into one of three mutually exclusive categories based on their foot characteristics: (i) one or both flat feet, (ii) one or both high arch feet, or (iii) both normal feet. Demographics, health characteristics, foot pain, and activity limitations were compared across these groups using chi-square tests (or Fisher's exact tests when expected cell counts were <5). Multivariable logistic regression was used to compare individuals with flat feet (versus normal) and individuals with high arch feet (versus normal), adjusting for age category, sex, race/ethnicity, educational attainment, income, current activity level, BMI, hypertension status, diabetes status, and arthritis status. The BMI categories used were based on standard definitions: underweight (<18.5 kg/m²), normal weight (18.5-24.9 kg/m²), overweight (25-29.9 kg/m²), and obese (≥30 kg/m²); due to small numbers, underweight and normal weight were combined for analysis. Assumptions for logistic regression were checked, and observations with missing data were excluded from that specific analysis. Data cleaning and analysis were performed using Stata versions 16.0 and 18.0 (StataCorp LLC, College Station, TX, USA) and R version 4.1.1 (R: A Language and Environment for Statistical Computing; R Foundation for Statistical Computing, Vienna, Austria). A two-sided p-value <0.05 was considered statistically significant, and p-values were adjusted for multiple comparisons using the Benjamini and Hochberg method.

## Results

**Figure 1:**
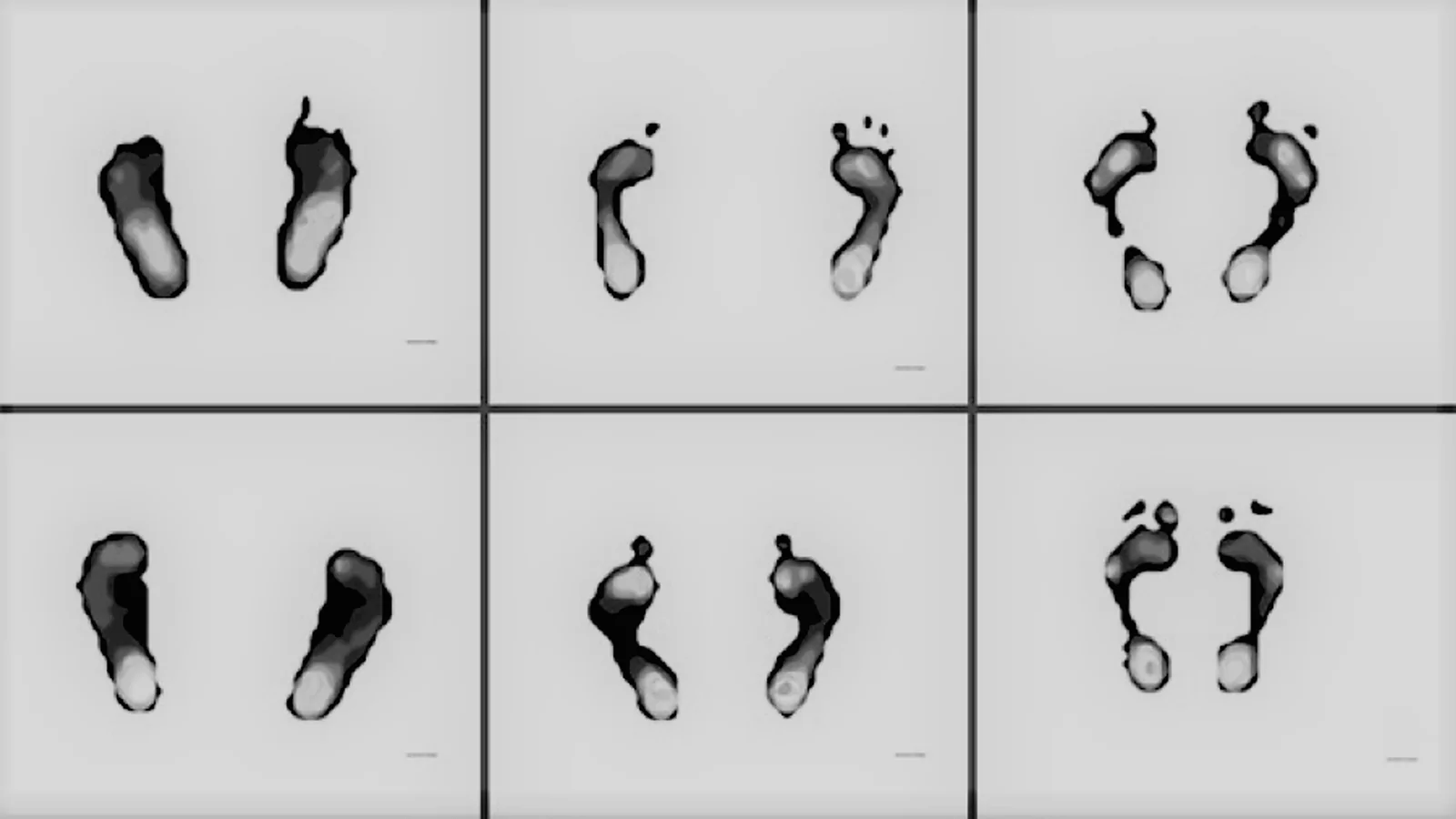
Representative plantar pressure profiles obtained using a Tekscan pressure mat from flat feet (left column), normal arch feet (middle column), and high-arch feet (right column).

Table 1 shows the distribution of flat, normal, and high arch feet by foot among study participants (n = 2,030). Flatfoot was slightly higher in the right foot compared to the left foot (83 (8.2%) versus 66 (6.5%)) among study participants; the opposite was true for high arch (Table 1). At the participant level, the overall prevalence of flatfoot (categorized as a participant having one or both flat feet) was 113 (11.1%) in the sample, with the overall prevalence of high arch feet (categorized as a participant having one or both high arch feet) being 186 (18.3%). Bilateral flat feet were observed in 36 (3.5%) of participants, while 88 (8.7%) had bilateral high arch feet; 77 (7.6%) and 98 (9.7%) had one flat or one high arch foot, respectively. The remaining 716 (70.5%) study participants had both feet categorized as normal; no participants had both one flat and one high arch foot. Figure 1 shows pressure profile examples for feet categorized as flat, normal, and high arch. Flat feet (left column) show more of the midfoot area touching the mat compared to those in the middle or right columns (Figure 1).

**Table 1:** Foot categorizations of study participants according to the mean distribution of the total surface area of the midfoot (n = 2,030 feet). ^a ^These results were generated using the log-transformed midfoot area (cm^2^) data. Flatfoot: Midfoot total area > Mean + 1 SD; High-arch foot: Midfoot total area < Mean - 1 SD; Normal: Midfoot total area is within -1 SD < Mean < +1 SD.

Foot	Flatfoot^a^		Normal foot^a^		High arch foot^a^	
	n	%	n	%	N	%
Right foot	83	8.2	800	78.8	132	13.0
Left foot	66	6.5	807	79.5	142	14.0

The sample was predominantly female (773 or 76.2%) and included a substantial proportion of African American participants (420 or 41.4%; Table 2). The highest number of study participants was in the 65-74 years age group (199 or 19.6%; Table 2). Significant demographic associations were observed for both flatfoot and high arch participant categories (Table 2). Flatfoot prevalence was highest in the 35 to 64 age ranges (20 or 11.2%, 23 or 12.6%, and 30 or 15.6% for 35-44, 45-54, and 55-65 age ranges, respectively, with a p-value = 0.028; Table 2). Males had higher flatfoot prevalence than females (47 or 20.6% vs 66 or 8.5%) but lower high arch prevalence (30 or 13.2% vs 152 or 19.7%; p = 0.0002). Flatfoot prevalence was highest among African Americans (83 or 19.8%) and lowest among Caucasians (24 or 4.7%; p = 0.0002), while the opposite pattern was observed for high arch (Caucasians: 150 or 29.1%; African Americans: 20 or 4.8%; p = 0.0002; Table 2). Flatfoot prevalence declined with increasing education (5 or 17.9% for less than high school diploma vs 18 or 8.5% for graduate/professional degree; p = 0.002), whereas high arch prevalence increased with education (3 or 10.7% vs 49 or 23.1% for less than high school diploma and graduate/professional degree, respectively; p = 0.002; Table 2). Income was not significantly associated with either category, though the association with flatfoot approached significance (Table 2).

**Table 2:** Distribution of sociodemographic and health-related characteristics by study participant foot categorization (n = 1,015). ^a ^p-values adjusted for multiple comparisons using the Benjamini and Hochberg method. ^b ^Other races include Asian, Biracial, Multiracial, Native Hawaiian or Other Pacific Islander, and Other.

Characteristics	Total		One or both flat feet		One or both high arch feet		Both normal		Χ^2^ test statistic	p-value^a^
	n	%	n	%	n	%	n	%		
Age Groups (years)									23.6642	0.028
18-24	49	4.8	4	8.2	15	30.6	30	61.2	-	
25-34	131	12.9	12	9.2	27	20.6	92	70.2		
35-44	179	17.6	20	11.2	23	12.9	136	76.0		
45-54	182	17.9	23	12.6	24	13.2	135	74.2		
55-64	192	18.9	30	15.6	30	15.6	132	68.8		
65-74	199	19.6	18	9.1	44	22.1	137	68.8		
≥75	74	7.3	6	8.1	20	27.0	48	64.9		
Missing	9	0.9	-	-	-	-	-	-		
Gender									27.6175	0.0002
Female	773	76.2	66	8.5	152	19.7	555	71.8	-	
Male	228	22.5	47	20.6	30	13.2	151	66.2		
Missing	14	1.4	-	-	-	-	-	-		
Race/Ethnicity									126.3906	0.0002
White or Caucasian	515	50.7	24	4.7	150	29.1	341	66.2	-	
Black or African American	420	41.4	83	19.8	20	4.8	317	75.5		
Hispanic	11	1.1	2	18.2	1	9.1	8	72.7		
Other Races^b^	42	4.1	4	9.5	8	19	30	71.4		
Refused	1	0.1	0	0	0	0	1	100.0		
Missing	26	2.6	-	-	-	-	-	-		
Educational Attainment									26.3644	0.002
Less than high school	28	2.8	5	17.9	3	10.7	20	71.4	-	
High school diploma/GED	300	29.6	41	13.7	44	14.7	215	71.7		
2-year college degree	212	20.9	26	12.3	24	11.3	162	76.4		
4-year college degree	261	25.7	22	8.4	66	25.3	173	66.3		
Graduate/Professional degree	212	20.9	18	8.5	49	23.1	145	68.4		
Missing	2	0.2	-	-	-	-	-	-		
Individual Income									20.9626	0.051
$24,999 or less	162	16.0	15	9.3	22	13.6	125	77.2	-	
$25,000 - $49,000	236	23.3	32	13.6	31	13.1	173	73.3		
$50,000 - $74,999	201	19.8	27	13.4	37	18.4	137	68.2		
$75,000 - $99,999	98	9.7	9	9.2	20	20.4	69	70.4		
$100,000 or more	111	10.9	9	8.1	31	27.9	71	64		
Other	31	3.1	1	3.2	6	19.4	24	77.4		
Unemployed	168	16.6	19	11.3	37	22	112	66.7		
Missing	8	0.8	-	-	-	-	-	-		
Body Mass Index (BMI)									222.851	0.0002
Underweight (<18.5 kg/m^2^)	9	0.9	0	0.0	7	77.8	2	22.2	-	
Normal Weight (18.5-24.9 kg/m^2^)	189	18.6	4	2.1	84	44.4	101	53.4		
Overweight (25-29 kg/m^2^)	247	24.3	9	3.6	62	25.1	176	71.3		
Obesity (≥30 kg/m^2^)	492	48.5	94	19.1	17	3.5	381	77.4		
Missing	78	7.7	-	-	-	-	-	-		
Current Activity Level									18.6934	0.0073
Inactive	42	4.1	6	14.3	7	16.7	29	69.0	-	
Slightly active	298	29.4	41	13.8	35	11.7	222	74.5		
Moderately active	465	45.8	47	10.1	90	19.4	328	70.5		
Very active	206	20.3	18	8.7	53	25.7	135	65.5		
Missing	4	0.4	-	-	-	-	-	-		
Hypertension									25.2637	0.0002
No	583	57.4	40	6.9	135	23.2	408	70.0	-	
Yes	401	39.5	70	17.5	44	11.0	287	71.6		
Missing	31	3.1	-	-	-	-	-	-		
Diabetes									25.2637	0.0002
No	807	79.5	78	9.7	167	20.7	562	69.6	-	
Yes	177	17.4	32	18.1	12	6.8	133	75.1		
Missing	31	3.1	-	-	-	-	-	-		
Arthritis									7.4932	0.028
No	705	69.5	67	9.5	127	18.0	511	72.5	-	
Yes	279	27.5	43	15.4	52	18.6	184	65.9		
Missing	31	3.1	-	-	-	-	-	-		

With regards to health-related characteristics, nearly half, or 492 (48.5%), of study participants were obese (Table 2). The majority, 969 (95.5%), reported any physical activity, with 206 (20.3%) stating they were very active (Table 2). Hypertension was reported by 401 (39.5%), diabetes by 177 (17.4%), and arthritis by 279 (27.5%; Table 2); 251 or 24.7% of study participants reported more than one of these conditions (data not shown). Flatfoot prevalence was significantly higher among obese participants compared to other BMI categories (94 or 19.1% vs 9 or 3.6% for overweight and 4 or 2.1% for normal/underweight; p-value = 0.0002; Table 2). In contrast, high arch feet prevalence was significantly higher in underweight/normal weight participants (7 or 77.8%; p = 0.0002) compared to overweight or obese study participants (Table 2). The presence of flat feet was also significantly higher in physically inactive study participants (6 or 14.3%; p = 0.0073) compared to those reporting any physical activity (Table 2). For chronic conditions, the prevalence of flatfoot was significantly higher for those with hypertension (70 or 17.5%; p = 0.0002), diabetes (32 or 18.1%; p = 0.0002), or arthritis (43 or 15.4%; p = 0.028; Table 2). While high arch followed opposite trends compared to flatfoot for both hypertension (44 or 11.0%) and diabetes (12 or 6.8%), this was not observed for arthritis (52 or 18.6%; Table 2).

Table 3 shows statistically significant adjusted odds ratios (AORs) from multivariable logistic regression models comparing flat feet and high arch feet to normal feet, adjusting for covariates as listed above. After adjustment, males had significantly higher odds of flat feet compared to females (AOR = 5.0, 95% CI: 2.8-8.9) and lower odds of high arch feet (AOR = 0.5, 95% CI: 0.3-0.9; Table 3). African American race was associated with significantly higher odds of flat feet (AOR = 5.7, 95% CI: 3.0-10.8) and lower odds of high arch feet (AOR = 0.2, 95% CI: 0.1-0.3) compared to Caucasians (Table 3). Other race was also associated with significantly higher odds of flat feet (AOR = 3.8, 95% CI: 1.2-12.3; Table 3). In addition, obesity was associated with increased flatfoot odds (AOR = 5.1, 95% CI: 1.7-15.6) and decreased high arch odds (AOR = 0.1, 95% CI: 0.0-0.1; Table 3). Arthritis also remained independently associated with flatfoot (AOR = 2.0, 95% CI: 1.2-3.6) but not with high arch (Table 3). Other covariates, including age, education, income, activity level, hypertension, and diabetes, were not significantly associated with either outcome in adjusted models (data not shown).

**Table 3:** Significant results (adjusted odds ratios (AORs) and 95% confidence intervals (CIs)) from multivariable logistic regression models for study participants with flat feet and high arch feet compared with normal feet. ^a^ Models adjusted for age, gender, race/ethnicity, educational attainment, income, current activity level, BMI, hypertension status, diabetes status, and arthritis status. *p-value < 0.05; **p-value < 0.001.

Characteristics	Flat Feet vs Normal Feet	High Arch Feet vs Normal Feet
	AOR^a^ (95% CI)	AOR^a^ (95% CI)
Gender		
Female	Reference Group	Reference Group
Male	5.0** (2.8-8.9)	0.5* (0.3-0.9)
Race/Ethnicity		
Caucasian	Reference Group	Reference Group
African American	5.7** (3.0-10.8)	0.2* (0.1-0.3)
Others	3.8* (1.2-12.3)	0.8 (0.3-2.0)
Body Mass Index (BMI)		
Underweight/Normal Weight (<25 kg/m^2^)	Reference Group	Reference Group
Overweight (25-29 kg/m^2^)	1.3 (0.4-4.4)	0.4* (0.3-0.7)
Obese (≥30 kg/m^2^)	5.1* (1.7-15.6)	0.1* (0.0-0.1)
Arthritis		
No	Reference Group	Reference Group
Yes	2.0* (1.2-3.6)	1.2 (0.7-2.0)

Participant-reported activity limitations and foot pain are shown in Table 4 for foot categories. Participants with flatfoot reported significantly higher percentages of activity limitation due to their feet (27 or 24.3%) compared with those with high arch (21 or 11.4%) or normal feet (154 or 21.6%; p-value = 0.007; Table 4). The ability to walk more than one mile comfortably also was significantly different across foot categorizations; only 54 or 47.8% of participants with flat feet could do so, compared with 141 or 75.8% of those with high arch feet and 428 or 60.0% of those with normal feet (p = 0.0002; Table 4). For ongoing pain, a higher percentage of flatfoot participants reported pain in at least one foot compared with high arch or normal foot participants (p = 0.022; Table 4). However, arch-specific pain did not differ significantly between groups (p = 0.051; Table 4). Notably, high arch participants consistently reported lower rates of pain and activity limitation than those with normal feet, a finding that contrasts with the pattern observed for flatfoot (Table 4).

**Table 4:** Activity limitation and pain by study participant foot categorization (n = 1,015). ^a^ p-values adjusted for multiple comparisons using the Benjamini and Hochberg method.

Characteristics	Total		One or both flat feet		One or both high arch feet		Both normal		Χ^2^ test statistic	p-value^a^
	n	%	n	%	n	%	n	%		
Activity limitation due to feet									10.8666	0.007
Yes	202	19.9	27	24.3	21	11.4	154	21.6	-	
No	807	79.5	84	75.7	163	88.6	560	78.4		
Missing	6	0.6	-	-	-	-	-	-		
Longest distance you can comfortably walk									33.8626	0.0002
≤1mile	389	38.3	59	52.2	45	24.2	285	40	-	
>1 mile	623	61.4	54	47.8	141	75.8	428	60		
Missing	3	0.3	-	-	-	-	-	-		
Ongoing pain in feet									15.5627	0.022
Yes, one foot	238	23.5	33	29.2	27	14.5	178	25.0	-	
Yes, both feet	179	17.7	22	19.5	21	11.3	136	19.1		
No	724	71.5	72	63.7	147	79.0	505	70.8		
Missing	3	0.3	-	-	-	-	-	-		
Ongoing pain in foot arch									9.1477	0.051
Yes, one arch	129	12.8	21	18.6	16	8.6	92	12.9	-	
Yes, both arches	28	2.8	4	3.5	4	2.2	20	2.8		
No/not sure	853	84.5	88	77.9	166	89.3	599	84.3		
Missing	5	0.5	-	-	-	-	-	-		

## Discussion

To our knowledge, this is among the first studies to use only pressure mat-derived midfoot total contact area to categorize flatfoot and high arch feet in a large, community-based adult sample and examine associations with participant characteristics, comorbidities, foot pain, and activity limitations. While the sample reflected the racial diversity of the study location, including a substantial portion of African American participants, it was a convenience sample that both overrepresented females and those aged ≥65 years and underrepresented younger individuals (<35 years of age).

We observed a flatfoot prevalence of 11.1% (at least one flat foot), which was lower than the 47.8% reported by López-López et al. [3], 33% reported by Debnath et al. [2], and 22.6% reported by Harris and Beath [19], but higher than the 2.6% reported by Yadav et al. [25], 3.3% by Kohls-Gatzoulis et al. [23], and 6.5% by Sun et al. [5]. This likely reflects differences in study populations (e.g., podiatrist clinic patients, military recruits, young adults <35 years of age, and females) compared with our population of community-based adults and measurement techniques (inked footprints compared with pressure mat). In addition, we classified foot arch using midfoot area only, while other studies employed methods such as Cavanaugh and Rodgers [29] or Brody’s navicular foot drop test [30]. Our classification method, therefore, provides a potential alternative to quickly and easily categorize foot arch morphologies.

As previously stated, other studies have reported a high arch prevalence of 5.2% to 49.6% [5,11,21,26]; as with flatfoot, our estimate of 18.3% (at least one high arch foot) falls within this previously reported broad range. However, most high arch prevalence estimates identified in the literature are lower than what we observed; only Sun et al. reported a higher prevalence from 127 women aged 18-25 years [5]. Direct comparisons are complicated by our use of a distribution-based categorization (±1 SD of the sample mean), which defines "abnormal" relative to the sample of feet rather than against an external clinical standard. Our high arch estimate may reflect the lower threshold for classification (±1 SD) that we used or differences in population characteristics.

Flatfoot prevalence peaked in the 55-64 years age group, consistent with studies showing increasing prevalence with age [20,22]. However, we observed a decline in flatfoot prevalence among participants ≥75 years. This pattern may reflect survival bias, age-related changes in foot mechanics, or measurement challenges in older adults and warrants further investigation. Our findings regarding race and sex align with prior reports, with African American participants and males showing significantly higher odds of flatfoot in adjusted models [22,28]. Obese participants also had significantly higher odds of flatfoot (AOR = 5.1), consistent with prior research [12]. However, our confidence interval associated with the obesity AOR is wide, suggesting a less precise estimate that should be interpreted cautiously. Even so, this observed association is likely multifactorial, reflecting both increased mechanical load on the foot and the higher prevalence of diabetes and related tendon dysfunction in obese populations. Notably, obesity was strongly inversely associated with high arch (AOR = 0.1), a pattern also observed for African American race and male sex. Other studies have not reported similar associations and have instead focused more on describing and predicting foot pain in those with high arches [5,11,21,26,27]. Hypertension, diabetes, and arthritis were each associated with flatfoot in unadjusted analyses, though only arthritis remained significant in adjusted models. The associations with hypertension and diabetes may potentially be mediated through obesity or other factors [4,8,20].

A notable finding of this study is the significant association between flatfoot and both activity limitations and general foot pain, an association that has not been consistently observed in prior research on asymptomatic participants. Badlissi et al. [27] found only a modest, non-significant association between flatfoot and pain in an older population (mean age 75), while Pita-Fernandez et al. [20] observed a lower but again non-significant reported quality of life in flatfoot participants. It is also noteworthy that the association we observed was significant for general foot pain but not for arch-specific pain, suggesting that flatfoot may contribute to overall foot discomfort rather than localized arch pain. Additionally, while flatfoot participants reported more pain and limitations, high arch participants reported less pain and limitations than those with normal feet; this counterintuitive finding contrasts with some prior studies [27]. Our null findings related to pain and high arch feet potentially correspond to Crosbie’s conclusion that higher arch severity did not equate to more pain [11]. The various biomechanical mechanisms underlying these associations between foot arch categorizations, pain, and activity limitation should be investigated further.

Pressure mat-based measurements may have utility in clinical and research settings for rapid, non-invasive foot arch assessment. Their efficiency and objectivity make this technology attractive for population-based studies and potentially for clinical screening [14-18]. However, further research is needed to validate the classification method used in this study with clinical examination and radiographic findings, calculate test-retest reliability of the pressure mat, and assess the clinical utility of identifying arch variations in asymptomatic individuals beyond communicating the burden and risk of these outcomes in the general population. Future studies should also investigate whether pressure mat findings predict diagnostic outcomes, guide treatment decisions, or reduce healthcare costs. Additionally, while we focused on static stance, dynamic gait analysis using pressure mats could provide complementary information about foot function during ambulation [11,14,16,18].

Several limitations warrant consideration. First, this was a convenience sample, which may limit generalizability and introduce selection bias. The sample was predominantly female (76.2%) and may not fully represent the broader population, despite being large (n = 1,015) and with participants recruited from multiple locations; we also do not have information on the total number of individuals approached. Second, and most importantly, our categorization of feet as flat, normal, or high arch was based on the distribution of midfoot contact areas within the sample itself (±1 SD of the mean). This approach meant that "normal" feet were categorized relative to the sample rather than through the use of an external clinical standard. Therefore, our calculated prevalence estimates are sample-dependent, limiting their direct comparison with studies using different definitions. Our results should, as stated previously, be validated against clinical examinations. Third, the cross-sectional design precludes causal inferences about the observed associations. Fourth, we did not collect information on prior conservative treatments or clinical foot and ankle diagnoses, which may have provided additional clinical context for the observed associations. Fifth, we did not assess test-retest reliability of the pressure mat measurements. Finally, the log-transformed threshold values may not be clinically meaningful, though appropriate for normalization of sample data. While estimating the prevalence of different arch classifications in a large sample was the main goal of this study, future studies can investigate these limiting factors.

## Conclusions

In this community-based sample, a flatfoot prevalence of 11.1% and a high arch prevalence of 18.3% were estimated using pressure mat-derived midfoot contact area. Flatfoot was associated with multiple demographics, including male sex, African American race, obesity, and arthritis. Notably, study participants categorized as having flat feet had significantly higher self-reported foot pain and activity limitations. Associations with high arch were also identified with female sex, Caucasian race, higher educational attainment, lower BMI, and arthritis. Higher percentages of high arch participants reported the ability to walk more than a mile and less pain. Given that our findings support what has been published in the literature, the pressure mat could be used in a clinical setting to potentially screen patients for foot arch variations, though validation of this methodology is necessary. In addition, with these baseline characteristics defined, clinicians can more confidently answer relevant patient questions around arch height and self-reported pain. Future research to assess natural arch changes, associated pathologies, and treatments may proceed with these baseline modern population-based estimates of the prevalence and diversity of adult foot arch contact.
